# Quantitative Trait Loci Identification and Candidate Genes Characterization for Indole-3-Carbinol Content in Seedlings of *Brassica napus*

**DOI:** 10.3390/ijms26020810

**Published:** 2025-01-19

**Authors:** Yiyi Xiong, Huaixin Li, Shipeng Fan, Yiran Ding, Mingli Wu, Jianjie He, Shuxiang Yan, Haibo Jia, Maoteng Li

**Affiliations:** 1 Department of Biotechnology, College of Life Science and Technology, Huazhong University of Science and Technology, Wuhan 430074, China; 2 Key Laboratory of Molecular Biophysics of the Ministry of Education, Wuhan 430074, China

**Keywords:** *Brassica napus*, QTL, indole-3-carbinol, glucosinolate, IGMT, molecular dockin

## Abstract

*Brassica napus* is a member of the cruciferous family with rich glucosinolate (GSL) content, particularly glucobrassicin (3-indolylmethyl glucosinolate, I3M), that can be metabolized into indole-3-carbinol (I3C), a compound with promising anticancer properties. To unravel the genetic mechanism influencing I3C content in rapeseed seedlings, a comprehensive study was undertaken with a doubled haploid (DH) population. By quantitative trait loci (QTL) mapping, seven QTL that were located on A01, A07, and C04 were identified, with the most significant contribution to phenotypic variation observed on chromosome A07 (11.78%). The genes within the QTL confidence intervals (CIs) include transcription factors (TFs) and glycosyltransferases. After co-expression analysis, GSL-related regulatory network of TFs-targets was constructed and two TFs, *BnaA07.ERF019* and *BnaA07.NAC92*, were identified as possible regulators in GSL biosynthesis. Three *IGMT* (*glucosinolate methyltransferases*) genes were found within the CIs that expressed higher in seedlings with more I3C, indicating their roles in I3C synthesis regulation. Molecular docking studies validated the binding capability of I3M to IGMTs, and those within the I3C QTL CIs have the strongest binding energy. These new discoveries offer critical insights into the genetic regulation of I3C content in rapeseed seedlings and establish a foundation for breeding high-I3C rapeseed varieties with potential health-promoting properties.

## 1. Introduction

*Brassica napus* (*B. napus*), a predominant oilseed crop in China, is not only a crucial source of edible oil but also a common edible vegetable, significantly contributing to various areas beyond oil production. Cruciferous vegetables are important in global nutrition, which are known for the abundance of chemopreventive compounds, such as glucosinolates (GSLs), polyphenols, and selenium. GSLs are a noteworthy class of secondary metabolites, which are especially prevalent in *Brassica* species and the model plant *Arabidopsis thaliana* (*A. thaliana*) [1], with over ninety types identified to date [2,3,4]. Studies have demonstrated that the consumption of cruciferous vegetables is associated with a reduced risk of several cancers, including lung [5], prostate [6], bladder [7], and gastric cancers [8]. The anticancer properties of these vegetables are largely due to the GSLs [9,10]. Among the GSLs, glucobrassicin (3-indolylmethyl glucosinolate, I3M) is the predominant indolic component [11], which can be converted into indole-3-carbinol (I3C) when hydrolyzed by the enzyme myrosinase. In previous studies, I3C has exhibited the inhibition ability of cancer cells with diverse mechanisms, including the induction of apoptosis in cancer cells [12], the inhibition of angiogenesis [13], and the induction of cell cycle arrest [14]. Furthermore, clinical studies of breast cancer and prostate cancer have found that I3C has not only therapeutic effects but also low toxicity [15]. This highlights the importance of GSLs, specifically I3M and its derivative I3C, in the potential cancer-preventive effects of cruciferous vegetables.

The biosynthesis of GSL mainly includes the elongation of the amino acid chain, the formation of the core structure, and the formation of side chains [16]. GSLs are derived from amino acids and thus can be classified into three groups according to their amino acid precursor, i.e., aliphatic GSLs (derived from amino acids of alanine, leucine, isoleucine, valine, and methionine), benzenic GSLs (derived from phenylalanine or tryptophan), and indolic GSLs (derived from tryptophan) [17]. Additionally, the transport and metabolism of GSLs also affect their form and levels in plants [18]. In *A. thaliana*, these series of processes had been extensively studied, and 99 related genes have been identified (www.arabidopsis.org, accessed on 2 July 2024). It was revealed that the indole glucosinolate methyltransferases (IGMTs) participated in the conversion of I3M to 1/4-methoxy-indol-3-yl-methyl (1/4MO-I3M) [19]. Researchers increased the expression level of *IGMT* by the overexpression of *IGMT*-responsive transcription factors (TFs) (such as *ERF6* and *WRKY33*), which could increase the content of 4OM-I3M and ultimately enhanced the disease resistance to *Botrytis cinerea* and *Alternaria brassicicola* [20,21]. Furthermore, the overexpression of *IGMT1-4* enhanced callose formation, whereas in *igmts* mutants, a significant reduction in callose formation was observed [22]. Moreover, *IGMT* genes also play a role in regulating the level of reactive oxygen species (ROS) in plants [22].

Quantitative trait loci (QTL) and a genome-wide association study (GWAS) were frequently used to analysis the genetic basis of GSL content in rapeseed. A study conducted in 2022 used a DH (double haploid) population with a high-density genetic map to identify 59 consensus QTL for seed GSL content, including four major QTL-HRs on chromosomes A09, C02, C07, and C09. Candidate genes, such as *BnaC09.SUR1* and *BnaC02.GTR2a*, related to sulfur assimilation and GSL transport were identified [23]. Liu et al. identified a major QTL *qGSL-C2* for seed GSL content that was located on chromosome C02 and could explain the 30.88–72.87% of the phenotypic variation observed in five environments, and they found that *BnaC2.MYB28* is likely associated with allelic variations correlated to GSL content [24]. GWAS analysis was also used to identify genetic loci that controlled the seed GSL content, and the loci on A07, A09, C02, and C09 were identified [25,26,27]. At present, only a few studies on the GSL content beside seeds in rapeseed were reported, the total contents of GSLs and 13 specific GSLs components in leaves and seeds within a DH population were measured, further QTL analysis was performed, and 105 key genetic loci were identified [28].

In the present study, QTL analysis was performed on I3C content of rapeseed seedlings by using a KN DH population, which was formed by doubling the haploid resulting from the hybridization of Ken-C8 and N53-2. Candidate genes were obtained within confidence intervals (CIs) of QTL through homologous genes alignment and co-expression analysis with BnIR TFs regulation network. As candidate genes, the expression patterns and potential functional domains of the *IGMTs* family genes were analyzed. Finally, the substrate binding activity of IGMTs was assessed by using molecular docking. The present study provides new insights into the genetic and metabolic controls of I3C content in the seedlings of rapeseeds.

## 2. Results

### 2.1. Detection of I3C Content in Seedlings of KN DH Population in B. napus

**Figure 1:**
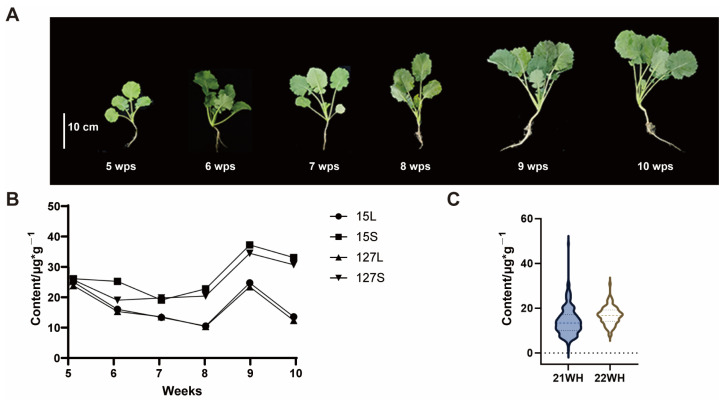
Detection of I3C content in seedlings. (**A**) Morphology of rapeseed seedlings at different harvest times; (**B**) I3C content in leaves (L) and stems (S) at different harvest times; (**C**) The distribution of I3C content in KN DH population.

Ten random samples were collected from the KN DH population two months after sowing to ascertain the content of I3C. These samples were divided into above-ground edible parts (stems and leaves) and underground roots for subsequent analysis. It was showed that the content of I3C in underground roots was relatively low (Figure S1). Subsequently, the seedlings of DH lines QT15 and QT127 with relatively higher I3C content were selected from 5 weeks post-sowing (5 wps) to 10 wps for further analysis. Throughout the sampling period from 5 wps to 10 wps, the results revealed that the I3C content was relatively high in the seeding, with an even greater concentration observed in the stems. Further analysis showed that the content of I3C started to decline gradually after 9 wps (Figure 1A,B).

Based on the above results, the seedlings of 9 wps were harvested for the I3C content analysis in successive experiments. It was revealed that the content of I3C varied a lot in different lines of KN DH population, and the content ranging from 1.538 μg/g to 48.769 μg/g in 2021 and from 7.954 μg/g to 31.181 μg/g in 2022, respectively (Figure 1C and Table S1). Further analysis indicated that the distribution of I3C content exhibited a normal distribution (Table S2), which was suitable for subsequent QTL analysis.

### 2.2. QTL Analysis of Seedling I3C Content in the KN DH Population

**Figure 2:**
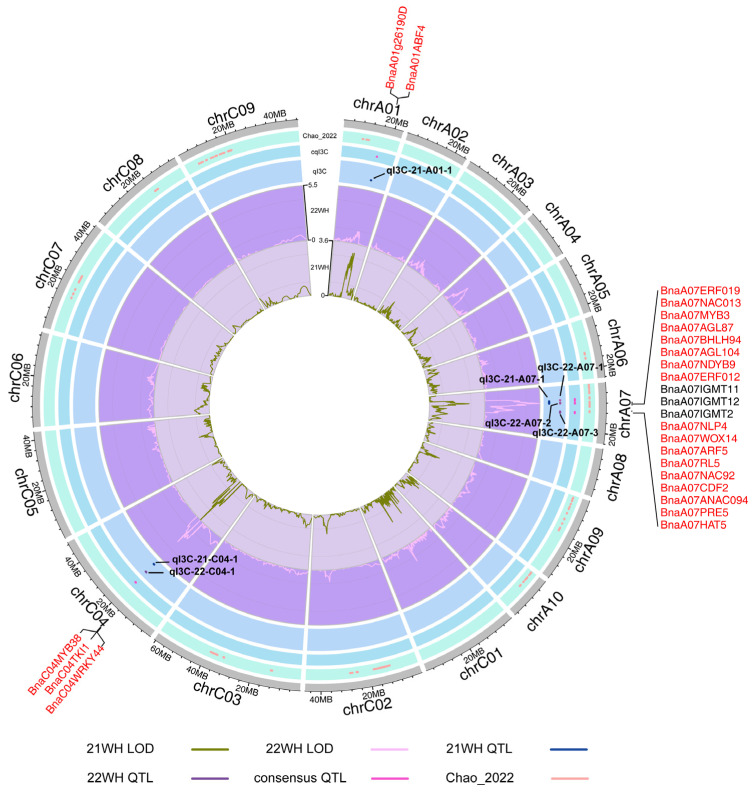
The distribution of all I3C QTL on 19 chromosomes, along with the related gene annotation within the CIs. The outermost layer is the names of the candidate genes within the QTL CIs. The inward layer is the physical length of chromosomes. In the circos diagram, from the outside to the inside, are the seed GSL content-related QTL reported by Chao et al. [23], the consensus I3C QTL, the I3C QTL identified in different years, and the LOD value distribution curves on each chromosome.

QTL mapping for the I3C content in seedlings of KN DH population was performed using the previously constructed high-density linkage map, and seven QTL were identified, respectively (Table 1 and Figure 2). Further analysis showed that three significant QTL located on chromosomes A01, A07, and C04 were detected in 2021, and the *qI3C-21-C04-1* had the highest phenotypic value of 6.65%. Four significant QTL that were located on chromosomes A07 and C04 were identified in 2022, and the *qI3C-22-A07-2* was had highest phenotypic value of 11.78% (Table 1 and Figure 2). In the same high-density genetic linkage map, meta-analysis can be used to examine the co-localization of different QTL for various traits, or QTL identified in different environments. Seven significant QTL were again integrated into five consensus QTL by meta-analysis, and only *cqI3C-A07-2* and *cqI3C-C04-3* were consistently mapped in both years, they were integrated from *qI3C-21-A07-1* and *qI3C-22-A07-2*, as well as *qI3C-21-C04-1* and *qI3C-22-C04-1*. Their CIs are located at 39.91–42.05 cM on chromosome A07 and 60.65–61.56 cM on chromosome C04, with respective phenotypic values of 8.25% and 6.53%.

Fifty-eight consensus QTL associated with the total seed GSL content were also obtained from the KN DH population in 2022 [23]. By integration analysis of the QTL obtained in this study and the QTL in the previous studies, it was found that *cqI3C-A01-1* and *cqI3C-C04-1* are the newly obtained QTL. *cqI3C-A07-1*, *cqI3C-A07-2* and *cqI3C-A07-3* are all co-located with the seed GSL content-related QTL. Part of the I3C QTL co-localized with GSL content-related QTL, which suggested that the contents of I3C and GSL in the KN DH population are regulated by the same genetic mechanisms to some extent. The newly identified I3C QTL implies that I3C is also regulated by partially independent genetic mechanisms.

### 2.3. The Candiate Genes Analysis Within CIs of QTL

**Figure 3:**
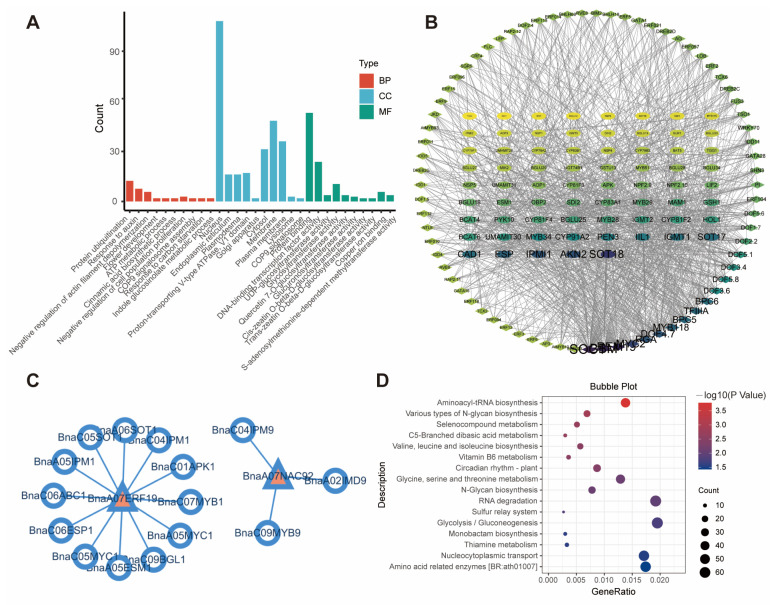
Functional analysis of genes in I3C QTL CIs. (**A**) GO enrichment analysis of all genes within the CIs of QTL, with different colors of bars representing different GO enrichment categories, BP for Biological Process, CC for Cellular Components, MF for Molecular Function. (**B**) Regulatory network of genes related to GSL synthesis and their potential upstream TFs in rapeseed, with TFs on the outer layer and functional genes on the inner layer—nodes with higher degree have deeper colors. (**C**) TFs within CIs and their potential regulatory genes, with TFs represented by triangles and functional genes represented by circles. (**D**) KEGG enrichment analysis of all genes potentially regulated by TFs within CIs, with the size and shape of bubbles representing the count value and *p*-value of different terms, respectively.

The candidate genes within all CIs of I3C-related QTL were predicted based on the covariance between genetic and physical mapping, and 287 genes within CIs were identified (Table S3). The gene ontology enrichment analysis showed that genes were mainly localized to the nucleus and endoplasmic reticulum within the cellular structure. And they played significant roles in biological processes such as protein ubiquitination and responses to auxins, with some genes directly involved in the metabolism of indolic GSLs (such as the *IGMTs* on A07). Notably, glycosyltransferase activity is the most enriched term in the molecular function category, along with protein and DNA binding TF activities. Since the biosynthesis of GSLs is partially dependent on glycosylation, these results suggest that the genes within the CIs may regulate the glycosylation steps in GSLs synthesis, thereby controlling the levels of I3M and total GSLs (Figure 3A, Table S4).

In order to analyze the GSL biosynthesis pathway in rapeseed, the GSLs synthesis-related genes from *A. thaliana* were collected. Ultimately, only three genes of the *IGMT* family (*BnaA07g11060D*, *BnaA07g11070D*, and *BnaA07g11080D*) within the CI of *qI3C-21-A07-1*, were found to be related to the metabolism of GSL. Further analysis showed that *BnaA07g11060D* and *BnaA07g11070D* are homologous to *IGMT1*, named *BnaA07.IGMT1.1* and *BnaA07.IGMT1.2*, respectively. *BnaA07g11080D* is a homolog of *IGMT2*, named *BnaA07.IGMT2*.

The research on the transcriptional regulation of GSL biosynthesis by TFs in rapeseed remains limited. In the present study, a co-expression analysis was performed focusing on 71 GSL-related genes from *Arabidopsis* as targets, using TF regulatory network module in BnIR (https://yanglab.hzau.edu.cn/BnIR/TF, accessed on 20 July 2024). The module obtained 1,506,425 TF-gene pairs based on the gene expression data of the ZS11 accession. A GSL-related regulatory network map of TFs-targets was constructed (Figure 3B). By analyzing the degree of all nodes in this network, it was found that TFs (such as AGAMOUS-LIKE 20 (SOC1), BABY BOOM (BBM), and REM19) emerged as regulators of multiple targets. Similarly, among the target genes, *SULPHOTRANSFERASE* (*SOT18*), *APS KINASE2* (*AKN2*), and *IPMI1* were all exhibited to a higher degree, which suggested that they were potentially regulated by several TFs (Figure S2).

Furthermore, 22 TFs were identified out of the 287 genes within all CIs (Table S5). Compared with TFs in BnIR, only *BnaA07.ERF019* and *BnaA07.NAC92* were found to have potential downstream co-expressed genes, with a total of 9167 genes potentially under their regulatory influence. These two TFs may directly regulate 15 coding genes involved in GSL synthesis and metabolism-related enzymes, including *BnaA05.IPM1*, *BnaC04.IPM9*, and *BnaA06.SOT1*, thereby playing a role in GSL content regulation (Figure 3C). KEGG enrichment analysis revealed that the functions of these genes are primarily associated with amino acid synthesis and metabolism (Figure 3D, Table S6). Additionally, pathways such as thiamine metabolism and the sulfur relay system that indirectly influence GSL biosynthesis through the regulation of sulfur elements were also found.

### 2.4. Identification and Characterization of IGMTs in Rapeseed

IGMTs participate in the conversion of I3M to 1/4-methoxy-indol-3-yl-methyl (1/4MO-I3M). A total of 17 *IGMT* family genes within the rapeseed genome were identified (Table 2), and the chromosome A07 harboring the most *IGMTs*, including the three genes *BnaA07.IGMT1.1*, *BnaA07.IGMT1.2*, and *BnaA07.IGMT2*, which were detected within the I3C QTL CIs. The coding sequence (CDS) lengths of these genes exhibit considerable variation, with *BnaA06g14960D* only having a CDS of 321 bp, while the majority of IGMTs have CDS lengths exceeding 1100 bp. ProtParam (https://web.expasy.org/protparam/, accessed on 8 October 2024) analysis revealed that the amino acid lengths range from 106 to 374 amino acids (aa), and the molecular weights vary from 11.77 to 41.21 kDa, indicating significant diversity at the amino acid level. Further analysis showed that the pI values range from 4.83 to 5.58, with an average of 5.05.

**Figure 4:**
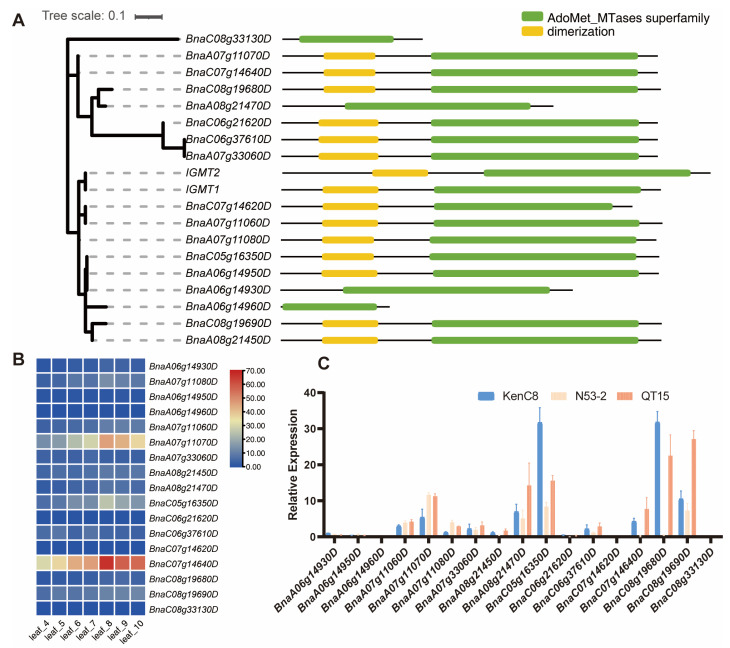
Analysis of the *IGMTs* family. (**A**) Evolutionary analysis and functional domain analysis of the *IGMTs* family genes, with the evolutionary tree on the left and the predicted domains from the amino acid sequences on the right. Different color blocks represent different domains, while straight lines indicate non-domain regions. (**B**) Expression levels of *IGMTs* family genes from the four-leaf stage to the ten-leaf stage, downloaded from BnIR. The color gradient from blue to red indicates increasing expression levels. (**C**) Validation of *IGMTs* expression levels by RT-qPCR.

A phylogenetic tree was constructed using the amino acid sequences translated from the 17 rapeseed *IGMTs* and two *Arabidopsis* homologous genes of *IGMT1* and *IGMT2* (Figure 4A). Their functional domains were predicted using the NCBI Batch CD-search tool (https://www.ncbi.nlm.nih.gov/Structure/bwrpsb/bwrpsb.cgi, accessed on 8 October 2024). It showed that the amino acid sequences translated from *IGMT1/2* in *Arabidopsis* all contain intact dimerization sites and methyltransferase functional domains. However, it was found that the IGMTs (BnaC08g33130D, BnaA08g21470D, BnaA06g14930D, and BnaA06g14960D) in *B. napus* have lost their dimerization sites, and some of which exhibit truncation in the methyltransferase functional domain, which might lead to the functional loss.

The gene expression analysis of 17 *IGMTs* from four-leaf stage to the ten-leaf stage was conducted, it revealed that some genes, including *BnaA06g14930D*, *BnaA06g14950D*, and *BnaA06g14960D*, displayed very low expression, while others (such as *BnaA07.IGMT1.1* and *BnaC07g14640D*) exhibited relatively higher expression (Figure 4B). Similar to the change in the I3C content, the expression of *IGMTs* were generally higher after the six-leaf stage and started to decline after the eight-leaf stage. Samplings of KenC8 and N53-2 (two parental lines of KN DH population) together with QT15 with higher I3C content at 8 wps were collected for further RT-qPCR analysis; the results showed that the target genes *BnaA07.IGMT1.1, BnaA07.IGMT1.2*, and *BnaA07.IGMT2* all presented a certain level of expression at this stage (Figure 4C), and their expression levels in N53-2 and QT15 with high I3C content were higher than that of KenC8 with low I3C content. This suggested that their expression levels may be the crucial factors to control the I3C content.

### 2.5. Molecular Docking of IGMTs in B. napus

**Figure 5:**
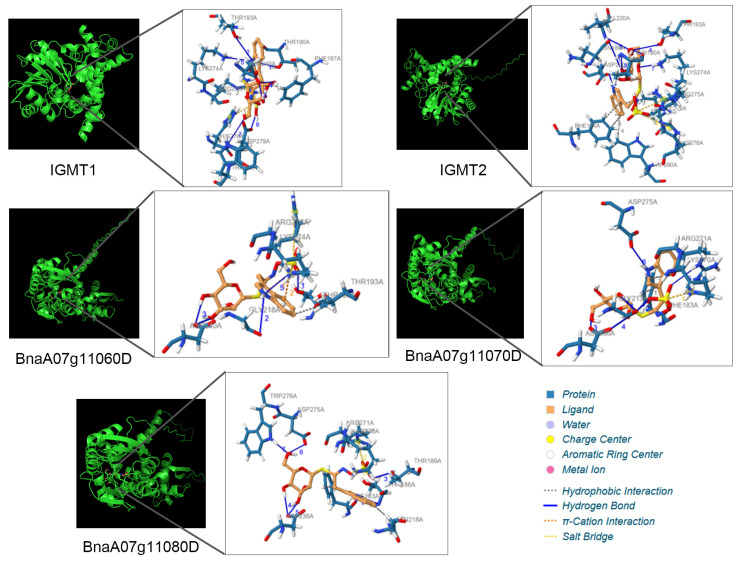
The binding mode of IGMTs with I3M, with the conformation of the highest binding energy selected. On the left is the overall structure of the protein–ligand complex, and on the right is an enlarged view of the binding site, with the amino acid residues involved in the interaction and the binding pattern indicated.

A small number of nucleotide differences can lead to amino acid sequence variations, which may result in the loss of the enzyme’s ability to bind to the substrate, ultimately leading to the loss of protein function. Therefore, to confirm the function of the *IGMTs* gene in *B. napus*, the molecular docking was used to verify the binding activity of the IGMTs protein to the substrate I3M. After eliminating genes with domain deletions and low expression levels, a total of 12 IGMTs, including IGMT1/2 from *A. thaliana*, were analyzed. AlphaFold 4 predicted protein structures served as receptors, with binding pocket locations identified using methyltransferase domain amino acid sequences from NCBI Batch CD-search. Meanwhile, the binding modes of three IGMTs within the I3C QTL region, as well as IGMT1/2 in *Arabidopsis*, with I3M were also analyzed (Table 3). Overall, their interactions with I3M mainly included hydrophobic interactions, hydrogen bonds, π-cation interactions, and salt bridges. Based on the analysis of the amino acid residues involved in the binding of the ligand molecule to the protein, taking the amino acids on IGMT1 as an example, it was indicated that the Phe187 was served as the object of hydrophobic binding in multiple ligand protein complexes, and showed a certain degree of conservation. Thr190, Thr193, Asp240, Lys274, and Asp279 are important amino acid residues that form hydrogen bonds with the ligand and also exhibit conservation in multiple complexes. In the process of salt bridge formation, Arg275 might play a major role (Figure 5, Table S7).

Using the binding energy of IGMT1/2 as a positive control, the binding energies of rapeseed IGMTs after docking with I3M were compared (Table 3). Initially, the binding energies of all IGMTs to I3M were analyzed. Among them, IGMT1 in *A. thaliana* exhibited the highest binding energy (−7.94 kcal/mol). In the IGMTs of rapeseed, BnaA07.IGMT1.2 and BnaA07.IGMT2 that were located within the CIs of the I3C QTL (*cqI3C-C04-1*) demonstrated the highest binding energies (reaching −6.65 and −6.77 kcal/mol, respectively), and both were higher than that of the binding ability of IGMT2 to the I3M in *A. thaliana*. The binding energy of BnaC06g37610D to I3M was the lowest (−5.32 kcal/mol). Notably, BnaC06g37610D also had the lowest expression level among the IGMTs used for molecular docking. These suggested that the higher binding ability and expression level of IGMTs within the I3C QTL CIs may ultimately make a significant contribution to the I3C phenotype.

## 3. Discussion

*Brassica* encompasses a variety of vegetables, including *B. nigra*, *B.oleracea*, *B.rapa*, *B. carinata*, *B. juncea*, and *B*. *napus*. These *Brassica* vegetables hold significant global economic value. *Brassica* plants are distinguished by their high nutritional content, featuring low levels of fat and protein, as well as abundant vitamins, fibers, and minerals [29]. Moreover, they are rich in phenolic compounds and contain a unique compound, GSL, which is also the difference between these crops and other vegetables [30]. The antimicrobial [31], antioxidant [32], and anticancer activities [33] of *Brassica* vegetables have been widely reported. In particular, the GSLs have been the subject of numerous studies for their anticancer potential [5,6,7,8]. Sulforaphane and I3M are prominent among the Brassica components, recognized for their potential anticancer effects. Sulforaphane is a kind of isothiocyanate, and previous studies showed that it has significant inhibitory effects on various types of cancer [34,35,36].

In the current research, the focus on I3M and its hydrolysis product, I3C, has largely been on their biological activities, with relatively fewer studies investigating their presence in plants. Rapeseed seedlings are notable for their tenderness in fiber and superior taste. This study selected the seedling stage, which is suitable for vegetable use, to measure the I3C content in the population and perform QTL mapping to elucidate the genetic mechanisms influencing I3C content during this period. Measured by Yu et al., the I3C content in the stems and leaves of rapeseed at the budding stage is 41.9 and 285.4 μg/kg, respectively, based on the fresh weight [37]. The I3C content in the rapeseed seedlings during the seedling stage could be more than tenfold higher than that in the rapeseed at the budding stage assuming that the dry weight is approximately 15% of the fresh weight [38]. The high-density genetic map of the KN DH population that was employed in this study was constructed by using SNP markers and additional types of markers [39,40]. In this study, seven QTL were identified, and the QTL for I3C on chromosomes A07 and C04 were co-located across the two years. In comparison to the previously reported QTL for seed GSL content, *cqI3C-A01-1* and *cqI3C-C04-3* identified in this study were first reported. In 2012, Feng et al., measured the contents of 15 different GSLs in rapeseed seeds and leaves. QTL mapping results revealed that the contents of different GSLs in seeds and leaves were mostly controlled by different loci [28]. Although I3C is the main hydrolysis product of indolic GSL in rapeseed, the QTL associated with I3C content in the KN DH population seedlings were co-localized with the seed GSL content-related QTL only on chromosome A07. These suggested that the I3C content in rapeseed seedlings is regulated by genetic mechanisms independent of the GSL metabolic pathway [23].

Due to the strong correlation between the high-density genetic linkage map and the physical map of the KN DH population used in this study, genes within the QTL CIs can be rapidly identified. GO enrichment analysis revealed a significant number of genes within these CIs are TFs, while the remaining genes are predominantly annotated as glycosyltransferases. It is known that glucosylation in GSL biosynthesis is primarily catalyzed by thiohydroximate S-glucosyltransferase [41]. However, subsequent research has uncovered that certain glycosyltransferases play a crucial role in the biosynthesis of GSLs. For instance, mutations in the *UDP-GLUCOSYL TRANSFERASE 74B1* (*UGT74B1*) gene in *A. thaliana* lead to a substantial reduction in GSLs. Moreover, these mutations resulted in the excessive accumulation of Indole-3-acetic acid, causing the plant to exhibit an auxin-overproduction phenotype [42]. Additionally, *UGT74C1* has also been shown to have similar functions to *UGT74B1*, being able to complement the phenotype and GSL content of *ugt74b1* mutants [43]. Furthermore, in contrast to the previously mentioned findings, some studies have identified that mutations in *UDP-glucose sterol glucosyltransferases* (*UGT80A2* and *UGT80B1*) in *Arabidopsis* result in an increased GSL level phenotype [44]. This suggests that the influence of glycosyltransferases on GSLs may involve more intricate mechanisms.

To investigate the association between TFs within the CIs of QTL for I3C, the BnIR database was utilized to input all genes related to GSL biosynthesis, thereby identifying TFs with co-expression relationships. Among these TFs, *BnaA07ERF19* and *BnaA07NAC92*, located within the I3C QTL CIs, were identified. Their potential downstream genes included homologous genes of *SOT1*, *ESM1*, and *BGL1*. In *Arabidopsis*, genes of the *SOTs* family, including *SOT1*, *SOT7*, *SOT16*, *SOT17*, and *SOT18*, encode the desulfoglucosinolate sulfotransferases [45]. These enzymes all played a crucial role in the final step of GSL biosynthesis by transferring the sulfate group to the precursor molecules [46]. Previous study revealed that all *SOTs* mutants exhibited significant phenotypes of decreased GSL levels [47]. *EPITHIOSPECIFIER MODIFIER 1* (*ESM1*) has been shown to mediate the conversion from I3M to indol-3-acetonitrile in *Arabidopsis* [48]. *BETA GLUCOSIDASE 1* (*BGL1*) has been identified as an endoplasmic reticulum marker protein that plays an important role in responses to abiotic stress [49]. Additionally, studies have found that *BGL1* is also involved in the conversion of I3M to indol-3-acetonitrile in *Arabidopsis* [50]. Furthermore, within the CIs of QTL, a total of 9167 downstream co-expressed genes were identified, which could be potential targets of transcriptional regulation. KEGG enrichment analysis of these genes revealed significant enrichment in pathways, such as thiamine metabolism and the sulfur relay system. These pathways are vital in the sulfur metabolism of plants, and sulfur deficiency can partially result in a decrease in GSL synthesis, which ultimately accelerates GSL degradation. Therefore, regulation of these pathways can indirectly affect the levels of GSLs [51].

The *IGMTs* encode a class of O-methyltransferase family proteins [52], which primarily control the conversion of I3M to 1OH-I3M and its subsequent methylation to transform into 1MO-I3M [19]. Previous studies have primarily focused on the role of IGMT and its related metabolites in plant defense against pests and pathogens [53,54,55]. The expression of *IGMTs* can be induced by various biotic and abiotic stress signals, such as γ-ray radiation [56], the pathogen *Botrytis cinerea* [20], and feeding by the green peach aphid, but can be inhibited by sucrose [57]. *IGMTs* have also been reported to be involved in the balance of ROS in *Arabidopsis*, ultimately affecting the formation of root callus [22]. Auxin is an important factor in the formation of callus, and callus serves as an essential material basis for resisting adversity stress [58]. However, under normal conditions, excessive callus accumulation can eventually lead to restricted plant growth. I3M has been proven to be another source of auxin [50], suggesting that the level of *IGMTs* in plants plays a crucial role in maintaining a stable level of I3M to control auxin levels and ultimately regulate the normal physiological functions of plants. Additionally, in some studies, there is a consistency between the increased expression levels of *IGMTs* and the elevated levels of I3M [59], three copies of the *IGMT* family genes within the CI of *cqI3C-A07-1* were found in the present study, and the higher expression levels of *IGMTs* that were observed in rapeseed were with high I3C content.

Molecular docking is a bioinformatics technique based on computer-aided design, which simulates the interaction between substrate molecules and protein active sites to predict the binding mode and binding energy between them [60]. Based on the level of binding energy, the affinity between the substrate and protein can be measured, and then the catalytic efficiency of the enzyme and the possible mechanism of action can be inferred. In plant research, molecular docking is used to determine the potential affinity of substrates for proteins, as well as to predict the binding strength of family proteins [61,62]. To further confirm the function of *IGMTs* in rapeseed, the genes with higher expression and complete domains through expression level analysis and domain analysis were screened out, followed by molecular docking to analyze the substrate binding activity of IGMTs. The results of the molecular docking revealed that the screened IGMTs all exhibited a certain substrate binding activity. Although the amino acid residues responsible for substrate binding are conserved, a few differences in the amino acid sequences can also lead to significant variations in the final binding capacity [63]. The *IGMTs* within the CIs of the I3C QTL showed the highest binding energies. Notably, the binding energy of BnaC06g37610D to I3M was the lowest, at only −5.32 kcal/mol. It is worth noting that the coding gene of BnaC06g37610D also had the lowest expression level among the *IGMTs* used for molecular docking. The same phenomenon has been found in the study of malate dehydrogenases in *Solanum lycopersicum* L. [64]. Homologous genes with better functional expression may have increased their expression levels through artificial selection in crops, but there is no direct evidence to prove the correlation between the binding ability and expression levels. This study suggests that the higher binding ability and expression level of *IGMTs* within the I3C QTL CIs may ultimately make a significant contribution to the I3C phenotype.

## 4. Materials and Methods

### 4.1. Plant Materials and Growing Condition

The KN DH population employed in this study was derived from the microspore culture of F1 plants, which resulted from the hybridization of Ken-C8 and N53-2 [39]. QTL analysis of I3C contents was performed using a genetic linkage map comprising 3106 SNP loci (encompassing 17,978 SNPs and 101 non-SNP markers, including SSR and STS) [39,40]. Field experiments were conducted in accordance with standard agricultural practices. Seeds harvested in 2021 and 2022 were utilized for the determination of I3C content.

### 4.2. Measurement of the I3C in Seedlings

The detection of I3C content was conducted following the experimental protocol described by Yu et al. in 2020 [37]. Briefly, rapeseed tissue was freeze-dried, thoroughly ground, and mixed evenly. A precise weight of 200 mg of sample powder was taken, to which pure water was added, followed by enzymatic hydrolysis at 45 °C for 2 h. The mixture was then centrifuged at 4 °C and 8000 rpm for 5 min. The supernatant was collected and extracted twice with dichloromethane. The extracted supernatant was purified using a C18 solid-phase extraction column to remove pigments and other impurities. The solutions were combined, dried under nitrogen, dissolved in acetonitrile, filtered through a 0.22 μm microporous filter membrane, and used for subsequent analysis. An Agilent 1200 high-performance liquid chromatograph equipped with an Agilent TC-C18 reverse-phase column (250 mm × 2.5 mm, 4.6 μm) and a VWD detector was used for detection (Agilent Technologies Inc., Waldbronn, Germany). The detection wavelength was set at 279 nm, with an injection volume of 20 μL and a column temperature of 30 °C. The mobile phase started at 90% water and 10% acetonitrile, gradually changing to 40% water and 60% acetonitrile over 20 min, followed by a complete switch to 100% acetonitrile, which was maintained until 30 min.

### 4.3. QTL Analyses and Candidate Gene Identification

QTL analysis was performed using Windows QTL Cartographer 2.5 software for composite interval mapping (CIM). The scanning step size was set at 1 cM, with a window size of 10 cM. The LOD threshold was determined through 1000 permutations to identify significant QTL, based on a 5% experimental error rate. All detected QTL were deemed significant and labeled accordingly. QTL integration was carried out using BioMercator 4.1 software [65]. The identified QTL were named by combining the phenotype, date, and chromosome number, for example, *qI3C-21-A07-1*, indicating the first I3C QTL on chromosome A07 identified in 2021. Consensus QTL were prefixed with “cq”, for example, cqI3C-A07-2, representing the second consensus QTL for I3C content on chromosome A07.

The alignment of genetic maps with physical maps and the identification of candidate genes were performed using the method described by Chao et al. [23]. Based on the collinearity between the high-density genetic map and the “Darmor-bzh” reference genome (http://www.genoscope.cns.fr/brassicanapus/data/, accessed on 9 April 2022), the genomic regions corresponding to the QTL CIs were determined using closely linked SNPs within the QTL CIs. Genes within these QTL CIs were considered to be candidates. These genes were annotated and named according to their homologous genes in *Arabidopsis* and the chromosome on which they were located (e.g., *BnaA07.IGMT1.1* represents the first orthologous gene of *IGMT1* on chromosome A07). The homologous genes were identified through BLASTn searches against the *Arabidopsis* database.

Genes related to GSLs content were gathered from the Gene Ontology database (http://geneontology.org/, accessed on 2 July 2024) by searching for terms such as “glucosinolate biosynthetic process”, “glucosinolate metabolic process”, and “glucosinolate transport process”. The collected GSLs-related genes were used to screen the candidate genes within the QTL CIs, aiming to identify the potential key target genes within these intervals. The QTL intervals and candidate genes were visualized using Circos software (version 0.69-9) [66].

### 4.4. Analysis of Genes Within the QTL CIs

The potential TFs of GSL-related genes were identified using the TF regulation network module of the BnIR website (http://yanglab.hzau.edu.cn/BnIR, accessed on 20 July 2024). Cytoscape software (version 3.9.0) was employed to construct a network of TFs and their functional target genes. The degree value for each node, representing the number of associations with other nodes, was obtained from Cytoscape. The enrichment analysis and visualization of KEGG and GO were conducted using the clusterProfiler and ggplot2 packages in R software (version 4.0.2) [67].

### 4.5. Sequence Alignment and Phylogenetic Analysis

The multiple sequence alignment of IGMTs proteins was conducted using the ClustalW program with its standard settings [68]. Subsequently, a neighbor-joining (NJ) method with the Poisson model was employed to construct phylogenetic trees. This process included a bootstrap analysis with 1000 replicates, utilizing MEGA 6 software.

### 4.6. Structure and Conserved Motif Analysis of IGMTs

The properties of IGMTs proteins were predicted using the ProtParam tool (https://www.expasy.org/, accessed on 8 October 2024), including the molecular weight and pI value of the proteins. Functional domains were identified using the Batch Web CD-Search Tools (https://www.ncbi.nlm.nih.gov/Structure/bwrpsb/bwrpsb.cgi, accessed on 8 October 2024) [69].

### 4.7. RT-qPCR of IGMTs in Rapeseed Seedlings

The first strand of cDNA was synthesized from the RNA using a HiScript III 1st Strand cDNA Synthesis Kit (Vazyme, Nanjing, China, R312). Each PCR reaction was conducted three times technically, following the instructions provided with the SYBR qPCR mix (Vazyme, Q712). Actin7 was used as a reference gene for normalization, and the analysis was performed using the QuantStudio 3 Real-Time PCR System (Thermo Fisher, Waltham, MA, USA), with each reaction performed in triplicate. The 2^−ΔΔCT^ method was employed to measure gene expression. The primers used for RT-qPCR are presented in Table S8.

### 4.8. Molecular Docking

Molecular docking was performed using AutoDock Vina. The proteins used for docking were all obtained from AlphaFold (https://alphafold.ebi.ac.uk/, accessed on 9 October 2024) [70]. After processes such as adding hydrogens, calculating charges, and modifying atom types in AutoDockTools, they were used as a Macromolecule for subsequent molecular docking experiments. I3M was obtained from the PubChem database and converted to the appropriate format using Open Babel software (version 3.1.0) [71]. It was then preprocessed with AutoDockTool (version 1.5.7) for use as a ligand in subsequent studies. The docking calculations were conducted for 50 iterations for each ligand, with the conformation exhibiting the lowest binding energy being identified as the most stable binder. Ultimately, the prediction of the interactions between the ligand-receptor complex was carried out using PLIP (https://plip-tool.biotec.tu-dresden.de/plip-web/plip/index, accessed on 10 October 2024) [72]. The properties of IGMTs proteins were predicted using the ProtParam tool (https://www.expasy.org/, accessed on 8 October 2024), including the molecular weight and pI value of the proteins. Functional domains were identified using the Batch Web CD-Search Tools (https://www.ncbi.nlm.nih.gov/Structure/bwrpsb/bwrpsb.cgi, accessed on 8 October 2024).

## 5. Conclusions

In this study, QTL mapping was utilized to identify genetic loci influencing I3C content in rapeseed seedlings. Furthermore, the potential IGMT genes that play a role in controlling the stable growth of high I3C content rapeseed plants were discovered. This research lays the groundwork for the subsequent cultivation of health-promoting rapeseed varieties with higher I3C content.

## Figures and Tables

**Table 1:** Summary of significant QTL detected for I3C content in 2021 and 2022.

QTL	Data	Chro.	Peak (cM)	CI (cM)	LOD	*R* ^2^	Genomic Region (Mb)
*qI3C-21-A01-1*	21WH	A01	72.81	72.6–73.1	3.00111	5.4751	18.22–18.31
*qI3C-21-A07-1*	21WH	A07	39.61	36.1–41.8	2.59966	4.7131	9.80–10.85
*qI3C-21-C04-1*	21WH	C04	61.11	61–62.2	3.61956	6.6496	6.00–6.51
*qI3C-22-A07-1*	22WH	A07	34.31	31.7–35.5	3.48936	7.8983	9.67–9.8.0
*qI3C-22-A07-2*	22WH	A07	41.21	39.4–41.7	5.48576	11.7816	10.55–10.85
*qI3C-22-A07-3*	22WH	A07	61.41	61.1–63.7	2.65608	5.5363	12.92–13.18
*qI3C-22-C04-1*	22WH	C04	61.11	60.9–62.3	3.12697	6.5276	6.00–6.51
consensus QTL							
*cqI3C-A01-1*		A01	72.81	72.6–73.1	3.00111	5.4751	18.22–18.31
*cqI3C-A07-1*		A07	40.98	39.91–42.05	2.59966	8.24735	10.56–11.08
*cqI3C-A07-2*		A07	41.21	39.4–41.7	5.48576	11.7816	10.55–10.85
*cqI3C-A07-3*		A07	61.41	61.1–63.7	2.65608	5.5363	12.92–13.18
*cqI3C-C04-1*		C04	61.11	60.65–61.56	4.243435	6.5276	6.00–6.51

**Table 2:** Summary of IGMTs family gene information in rapeseed.

Darmor Gene ID	At ID	Chr.	Position	CDS (bp)	Size (aa)	Molecular Weight (kDa)	pI
*BnaA06g14930D*	*AT1G21100*	A06	8,165,790..8,166,940	864	287	31.57	5.42
*BnaA06g14950D*	*AT1G21100*	A06	8,171,265..8,174,191	1122	373	40.98	5.01
*BnaA06g14960D*	*AT1G21100*	A06	8,175,856..8,176,272	321	106	11.77	5.08
*BnaA07g11060D*	*AT1G21130*	A07	10,417,318..10,418,638	1128	375	41.20	5.11
*BnaA07g11070D*	*AT1G21100*	A07	10,420,377..10,421,893	1110	369	40.33	4.97
*BnaA07g11080D*	*AT1G21120*	A07	10,425,492..10,426,829	1110	369	40.52	4.98
*BnaA07g33060D*	*AT1G76790*	A07	22,741,269..22,743,280	1113	370	40.98	4.99
*BnaA08g21450D*	*AT1G21120*	A08	15,882,392..15,883,701	1125	374	41.21	4.86
*BnaA08g21470D*	*AT1G21130*	A08	15,887,541..15,888,917	801	266	29.45	5.07
*BnaC05g16350D*	*AT1G21120*	C05	10,162,344..10,163,735	1119	372	40.63	4.97
*BnaC06g21620D*	*AT1G76790*	C06	23,825,989..23,827,885	1113	370	40.97	4.83
*BnaC06g37610D*	*AT1G76790*	C06	35,541,776..35,543,789	1113	370	41.00	4.93
*BnaC07g14620D*	*AT1G21120*	C07	20,535,358..20,536,661	1119	372	38.28	4.99
*BnaC07g14640D*	*AT1G21100*	C07	20,540,694..20,541,985	1110	369	40.28	4.91
*BnaC08g19680D*	*AT1G21100*	C08	22,527,597..22,529,146	1119	372	40.84	5.21
*BnaC08g19690D*	*AT1G21120*	C08	22,539,023..22,540,323	1125	374	41.24	4.90
*BnaC08g33130D*	*AT1G21100*	C08	31,741,406..31,742,062	414	137	15.11	5.58

**Table 3:** The location of the methyltransferase domain in IGMTs, the molecular docking pocket location, and the binding energy.

Targets Protein	MT Domain		Binding Pocket			Binding Energy
	Start	End	X Axis	Y Axis	Z Axis	
BnaA07g11060D	150	353	−6.821	−0.275	5.486	−6.06
BnaA07g11070D	146	350	−6.684	0.076	7.066	−6.65
BnaA07g11080D	146	350	−6.567	−0.860	7.070	−6.77
BnaA07g33060D	147	352	0.723	−2.120	−0.333	−5.53
BnaA08g21450D	148	352	−6.634	−0.165	6.937	−6.35
BnaC05g16350D	146	350	−6.205	−1.572	6.435	−5.5
BnaC06g37610D	147	352	−7.558	0.296	7.693	−5.32
BnaC07g14640D	146	350	−6.652	−0.586	6.703	−6.18
BnaC08g19680D	146	350	−6.819	−0.598	6.967	−6.13
BnaC08g19690D	148	352	−7.025	−0.114	6.826	−6.29
IGMT1	150	354	−4.607	−2.628	9.389	−7.94
IGMT2	198	402	−4.179	−2.418	9.022	−6.33

## Data Availability

Data are contained within the article.

## Supplementary Materials

The following supporting information can be downloaded at: https://www.mdpi.com/article/10.3390/ijms26020810/s1.
