# Supplementary material for: Quantitative Trait Loci Identification and Candidate Genes Characterization for Indole-3-Carbinol Content in Seedlings of Brassica napus
Source: Int J Mol Sci. 2025 Jan 19;26(2):810. doi: 10.3390/ijms26020810 (PMC11766266; doi:10.3390/ijms26020810)
Supplement: Supplementary file 1 [file ijms-26-00810-s001.zip › Supplementary Figures.pdf]

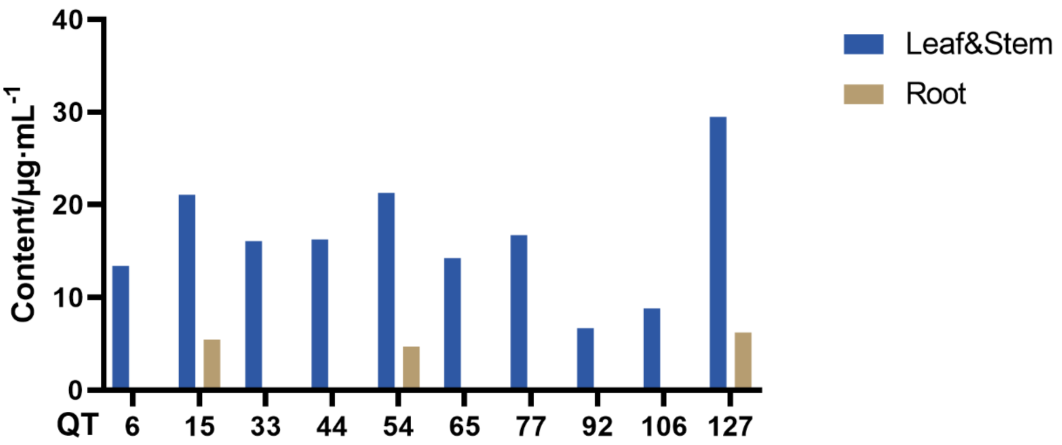

Figure S1. In 2020, 10 plants were randomly sampled from the KN DH population, and the I3C content in the above-ground parts (leaves, stems) and underground parts (roots) of rapeseed seedlings was detected.

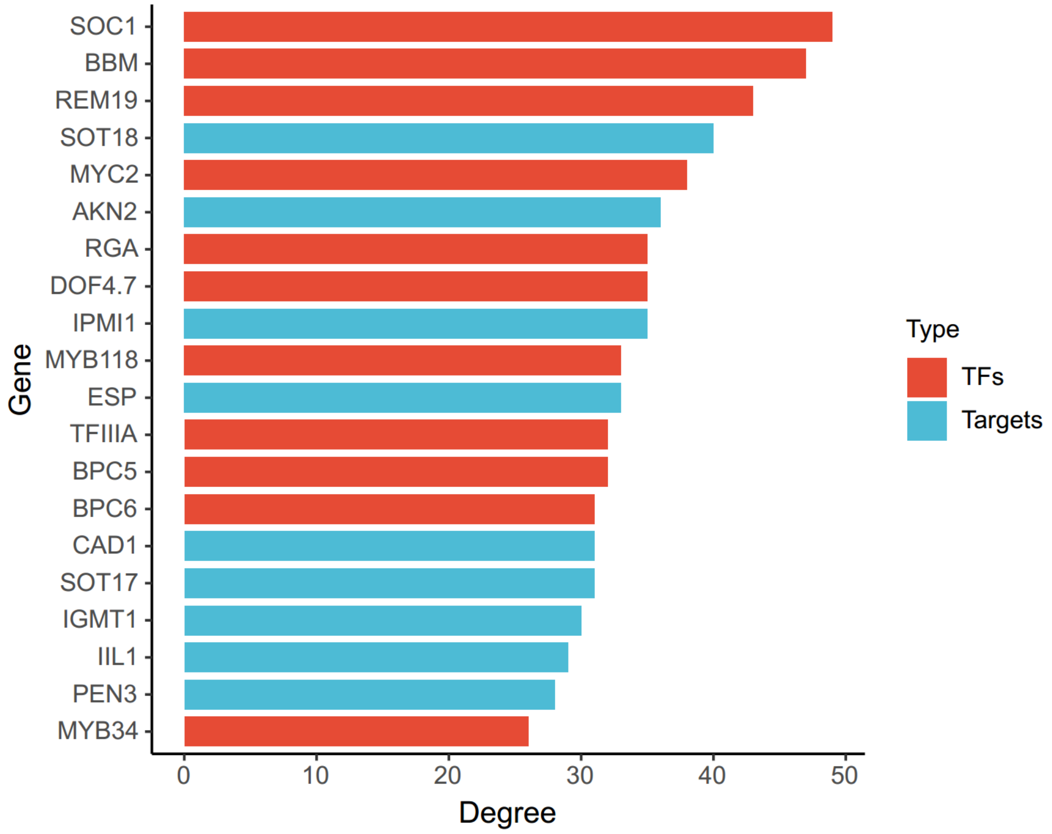

Figure S2. The degree of each node in the GSLs-related gene-transcription factor network. Bars of different colors represent different types of genes.
